# Recurrence of lymphoma with isolated pericardial mass: a case report

**DOI:** 10.1186/s13256-020-02508-4

**Published:** 2020-10-03

**Authors:** Istemi Serin, Avni Ulusoy, Mediha Irem Onar, Mehmet Hilmi Dogu

**Affiliations:** 1 Department of Hematology Istanbul, University of Health Sciences, Istanbul Training and Research Hospital, 34098 Istanbul, Turkey; 2 Department of Internal Medicine, University of Health Sciences, Bagcilar Training and Research Hospital, Istanbul, Turkey

**Keywords:** Diffuse large B-cell lymphoma, Recurrence, Extranodal recurrence, Pericardium, Case report

## Abstract

**Background:**

Diffuse large B-cell lymphoma is the most common subtype of non-Hodgkin lymphoma and may occur with lymph node and/or extranodal involvement. Recurrence in patients with diffuse large B-cell lymphoma usually occurs within the first few years after treatment and may occur in a different area outside the initial localization.

**Case presentation:**

A female Turkish patient who was diagnosed with nodular sclerosing Hodgkin lymphoma through lymphadenopathy examination reached remission after chemotherapy and radiotherapy. In the 11th year of follow-up and at the age of 45, newly developed multiple lymphadenopathies were diagnosed with a pathological result of diffuse large B-cell lymphoma in her advanced examination. Due to massive splenomegaly and cystic necrotic splenic residues, splenectomy was performed after eight cycles of a first-line chemotherapy regimen and two cycles of high-dose methotrexate treatment for central nervous system prophylaxis. A pericardial mass (maximum standardized uptake value 34.8), which was not present at the time of diagnosis and interim evaluation of positron emission tomography/computed tomography, was detected through chest pain in the third month after the last screening, although a complete response had been obtained. Pathological examination of the pericardial area revealed the pathological result was a recurrence.

**Conclusions:**

Patients with diffuse large B-cell lymphoma have an aggressive clinical course, but cardiac involvement is very rare. In our patient’s case, pericardial involvement was observed after treatment and scanning revealed that recurrence took place in an area different from the pericardium. Cooperation of clinicians and pathologists and rapid evaluation are very important in cases of diffuse large B-cell lymphoma relapse. Although a tumoral invasion of the pericardium mostly suggests secondary malignancies, it should be kept in mind that recurrence of lymphoma is also possible.

## Introduction

Diffuse large B-cell lymphoma (DLBCL) is the most common subtype among non-Hodgkin lymphomas (NHLs). It accounts for about 30–40% of NHL cases and more than 80% of aggressive lymphomas [1]. Like many other NHLs, it is more common in men, with about 55% of cases [2]. Incidence increases with age; the median age is 64 [3]. DLBCL, a clinically aggressive lymphoma, may occur in lymph node and/or extranodal areas. The most common areas of extranodal involvement are gastrointestinal tract, skin, bone marrow, or paranasal sinuses [4, 5]. Patients typically present with a rapidly growing symptomatic mass or painless lymphadenopathies that develop in the cervical, supraclavicular, or axillary areas. Systemic “B” symptoms (fever, weight loss, night sweats) occur in about 30% of patients and are considered as a bad prognosis [6]. Treatment in patients with DLBCL also depends on the precise histological subtype, the extent of the disease, its localization, and the patient’s performance. Advanced disease is defined as stage III or IV according to the Ann Arbor classification and accounts for approximately 70% of patients with DLBCL. A combined recombinant anti-CD20 antibody, systemic chemotherapies, and involvement radiotherapy are the most distinguished treatments when DLBCL is diagnosed. Long-term disease-free survival in patients with DLBCL occurs in at least 50% of patients, and when lymphoma is localized at diagnosis, it may go up to more than 80% of these patients [7, 8]. In patients in remission, relapse usually occurs within the first 2–3 years after treatment. Relapse that occurs 5 years after treatment is called “late relapse” and is rarer [9]. Relapse may also occur in a different area from the first localization [10]. In this case report, we present a case of a patient who has received chemoradiotherapy due to nodular sclerosing Hodgkin lymphoma (HL) in the past and who has been diagnosed with NHL after more than 10 years, with isolated pericardial recurrence after treatment.

**Fig. 1:**
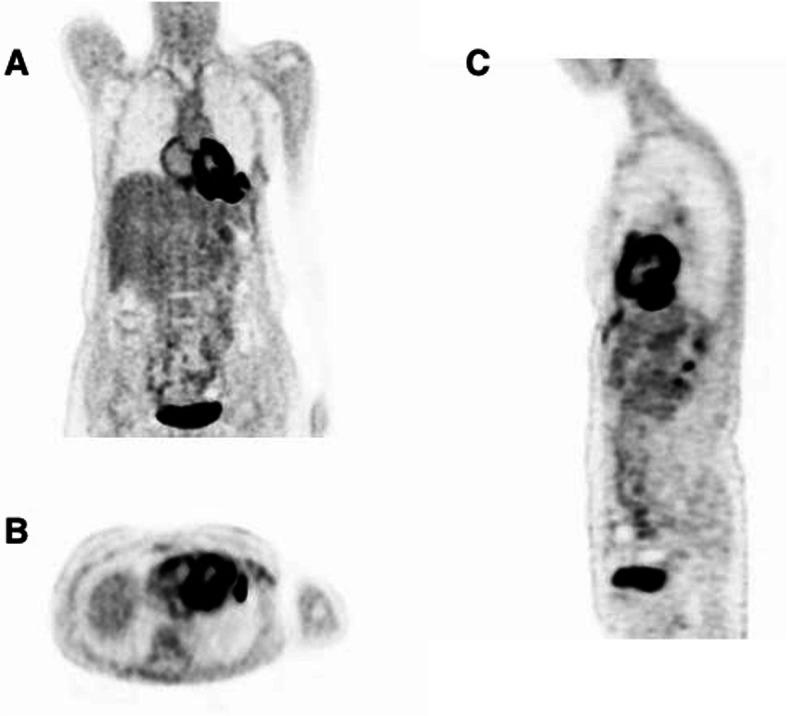
Coronal (**a**), transverse (**b**), and sagittal (**c**) positron emission tomographic images of pericardial mass

**Fig. 2:**
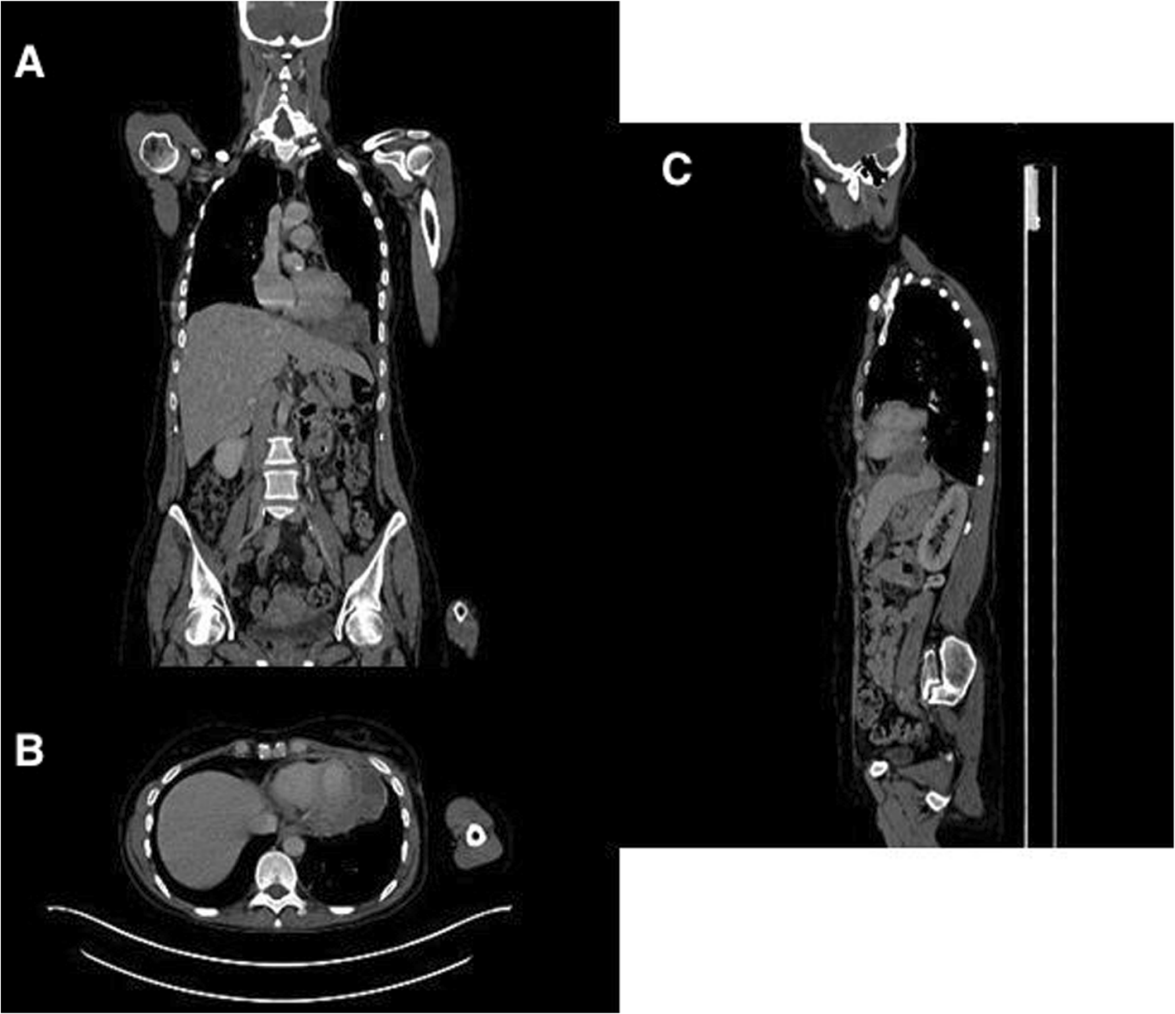
Coronal (**a**), transverse (**b**), and sagittal (**c**) computed tomographic images of pericardial mass

## Case presentation

A female Turkish patient who was diagnosed with nodular sclerosing HL by being examined with lymphadenopathy reached remission after receiving an ABVD (doxorubicin, bleomycin, vinblastine, dacarbazine) chemotherapy regimen for three cycles and 30-Gy radiotherapy. In the 11th year of follow-up and at the age of 45, newly developed multiple lymphadenopathies were diagnosed with a pathological result of DLBCL in her advanced examination. Laboratory test results of the patient were as follows: hemoglobin 8.8 g/dl, white blood cell 11,620/mm^3^, neutrophil count 8540/mm^3^, platelet count 456,000/mm^3^, urea 22 mg/dl, creatinine 0.64 mg/dl, uric acid 7.1 mg/dl, aspartate transaminase 25 U/L, alanine transaminase 13 U/L, lactate dehydrogenase 848 U/L, total protein 6.4 g/dl, albumin 3.2 g/dl, and C-reactive protein 68 mg/dl. Positron emission tomography/computed tomography (PET/CT) showed hypermetabolic fluorodeoxyglucose (FDG) involvement in the bilateral jugular chain in the neck (maximum standardized uptake value [SUVmax] 14.8), spleen sizes in the abdominopelvic sections (270 mm), hypermetabolic FDG in places of heterogeneous character in this cystic lesion area, the largest of which reaches a diameter of 70 mm (SUVmax 30.7), hypermetabolic FDG involvement (SUVmax 14.0) in the para-aortocaval region, hepatogastric area, bilateral inguinal fossa at the portal hilus level, and sclerotic lesion in the midline in the sacrum (SUVmax 15.8). The patient was diagnosed with DLBCL. Immunohistochemistry results were as follows; Ki-67 80%, CD20 (+), BCL2 (+), BCL6 (+), MUM1 (+), C-MYC (−), nongerminal center type DLBCL, and fluorescence in situ hybridization negative for c-myc by cervical lymph node biopsy. In addition to eight cycles of R-CHOP (rituximab, cyclophosphamide, doxorubicin, vincristine, prednisone) treatment, the patient received central nervous system prophylaxis with two cycles of high-dose methotrexate because of her high central nervous system international prognostic index score (a total score of 5, indicating high risk). Doxorubicin was also removed from the protocol after reaching the cumulative dose of 450 mg/m^2^. The pathology of the splenectomy specimen did not include tumoral invasion; only a cystic necrotic area was observed. At the end of treatment, PET/CT was performed 1 month after surgery and was compatible with complete remission.

Three months after the last PET/CT control, she was admitted to our outpatient clinic due to chest pain and B symptoms. A hypermetabolic mass, which was evaluated in favor of the involvement of primary disease, was observed isolated in the widest place in contact with the pericardium at the left precardial distance 2.9 × 2.2 cm (SUVmax 34.8) (Figs. 1 and 2), although there was no sign of recurrence in the interim or at the end of treatment on the basis of PET/CT scanning. A pericardial biopsy was performed for possible secondary malignancy due to the absence of involvement in another area of ​​the whole body, and it was found to be compatible with CD20 (+), BCL2 (+), BCL6 (+), C-MYC (−) immunoreactivity nongerminal center type DLBCL recurrence. The results of Ehrlich-Ziehl-Neelsen staining and Löwenstein-Jensen cultures were negative for tuberculosis.

## Discussion

Primary tumors of the pericardium are extremely rare. The first malignancies that come to mind in the presence of pericardial effusion and especially tamponade are lung cancer with 30% and breast cancer with 20% [11, 12]. The patients are diagnosed through pericardial sampling and biopsies. Especially, as in our patient’s case, the development of secondary malignancies in the group of patients who have received many cytotoxic treatments and radiotherapy has been increasing in number, so are lung and breast malignancy treatments in the clinic. The emergence of a clinical finding with a high cytotoxic treatment burden and not at the beginning of treatment and not observed in interim imaging at post-treatment follow-up suggested a new malignancy in our patient’s case.

Cardiac and pericardial involvement of lymphoma is extremely rare, accounting for 0.5% of cardiac involvement and 1% of extranodal NHL. The most common lymphoma subtype is known to be DLBCL, but Burkitt lymphoma, T-cell lymphomas, small lymphocytic lymphoma, and plasmablastic lymphoma also occur [13]. The formation of a pericardial effusion or mass can occur with hematogenous spread as well as the direct spread of lymphoma directly. Massive pericardial effusion, in particular, is seen in two rare NHL subtypes: primary cardiac lymphoma (PCL) and primary effusional lymphoma (PEL). PCLs are a rare subtype of lymphoma predominantly present in male patients. The incidence of PCL has been on the rise in relation with patients with acquired immunodeficiency syndrome and transplant recipients receiving immunosuppressive therapy. PCL usually presents after the fifth decade of life and most often involves the right heart chambers. Heart failure and pericardial effusions are the two most commonly reported clinical presentations of PCL. Patients apply to the clinic most frequently with dyspnea [13]. PEL is an NHL subtype that occurs also in patients with human immunodeficiency virus infection, especially in the body cavities without a mass form [14].

Primary mediastinal large B-cell lymphoma accounts for 7% of DLBCLs and represents 2.4% of NHL cases. The disease is observed especially in the third and fourth decades and predominantly in women. Tumor compression or invasion, especially of anterior mediastinal origin, is observed. Isolated pericardial mass or effusion is not expected [15].

Involvement-related arrhythmia may occur with many clinical findings such as shortness of breath and syncope. It usually indicates a patient with lower chance of survival. It is more common in high-grade lymphomas, especially double-hit/triple-hit subtypes, and shows a poor prognosis. Although it is mostly seen with cardiac symptoms, it can also occur asymptomatically. Generally, clinical manifestation occurs with right ventricular dysfunction and tamponade, which also carry important clues for central nervous system involvement [16].

Our present case report describes a very rare condition because it is about a patient with DLBCL long after HL-specific treatment and recurrence with an isolated pericardial mass after DLBCL treatment. Although the literature indicates that lymphomas with isolated cardiac recurrence or cardiac involvement were particularly associated with acquired immunodeficiency syndrome, our patient’s case does not have such comorbidity. Although isolated pericardial mass suggested secondary malignancies, pathology revealed recurrence of isolated DLBCL recurrence.

## Conclusions

Pericardial involvement is rarely seen among lymphomas, and DLBCL is the most common subtype of them. Although nonlymphoma tumors first come to mind in patients with a history of cytotoxic therapy anamnesis and in cases that are at risk for secondary malignancy, it should be kept in mind especially in high-grade lymphomas in terms of recurrence.

## Data Availability

Data are included in this published article and its additional file.

## Abbreviations

DLBCL: Diffuse large B-cell lymphoma
FDG: Fluorodeoxyglucose
HL: Hodgkin lymphoma
NHL: Non-Hodgkin lymphoma
PCL: Primary cardiac lymphoma
PEL: Primary effusional lymphoma
PET/CT: Positron emission tomography/computed tomography
R-CHOP: Rituximab, cyclophosphamide, doxorubicin, vincristine, prednisone
SUVmax: Maximum standardized uptake value
